# Supplementary material for: A Green Synthesis Strategy for Cobalt Phosphide Deposited on N, P Co-Doped Graphene for Efficient Hydrogen Evolution
Source: Materials (Basel). 2023 Sep 7;16(18):6119. doi: 10.3390/ma16186119 (PMC10532637; doi:10.3390/ma16186119)
Supplement: Supplementary file 1 [file materials-16-06119-s001.zip › materials-2506705-supplementary.pdf]

DNA was placed in a quartz boar in a tubular furnace and heated to 800 °C at a heating rate of 5 °C $min^{-1}$ in an Ar atmosphere before being kept for 2 h. After washing and filtering with deionized water, the NPC composite was obtained by vacuum drying overnight. XPS was used to verify the detect the element composition and chemical states of NPC. As shown in Figure S1a, the peaks can be divided into four peaks at 284.8, 285.7, 286.7, and 288.8 eV, contributing to the C-C, C-P, C-N, and C-O, respectively. The high-resolution spectrum of N 1s (Figure S1b) can be deconvoluted into two peaks, which can be ascribed to pyridinic N (398.4 eV) and graphitic N (401.2 eV), indicating the successful doping of N to the carbon. For P 2p (Figure S1C), two peaks located at 133.1 and 134.0 eV can be ascribed to P-C and P-O species. All of the results above demonstrate that the DNA molecules transfer to N, p co-doped carbon (NPC) after the carbonization process.

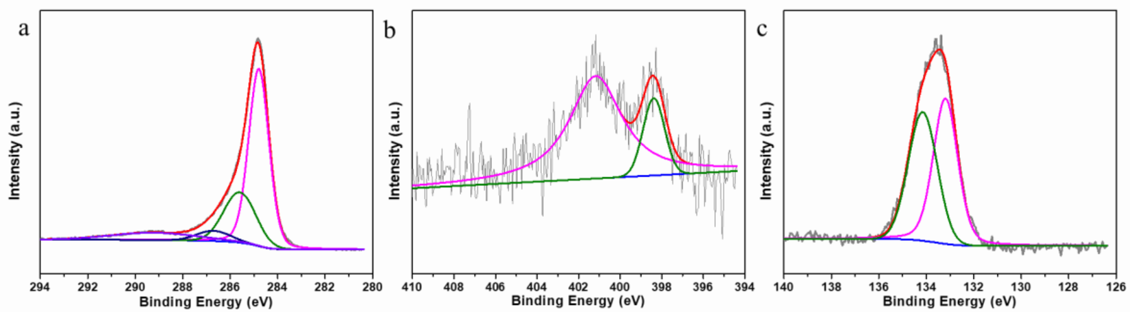


**Figure S1.** High-resolution XPS spectra of C 1s (a), N 1s (b), and P 2p (c) for NPC.

**Table S1.** Comparison of catalytic performance with other non-noble metal catalysts.

| Catalyst | $\eta_{10}$ (mV) | reference |
|---|---|---|
| CoP-$V_4P_3$-CNT | 124 | [1] |
| Cu-CoP NF | 170 | [2] |
| $Co_2P$/WC@NC/CNTs | 171 | [3] |
| C/Fe-$CoS_2$ | 151 | [4] |
| CoP@NCNWs/CF | 85 | [5] |
| $Co_2P$@BNC | 75 | [6] |
| CoP/NCNHP | 140 | [7] |
| $Ni_2P$/$Co_2P$@NC | 226 | [8] |
| $Co_2P$@NPC | 176 | [9] |
| $Co_2P$/NPG | 144 | This work |

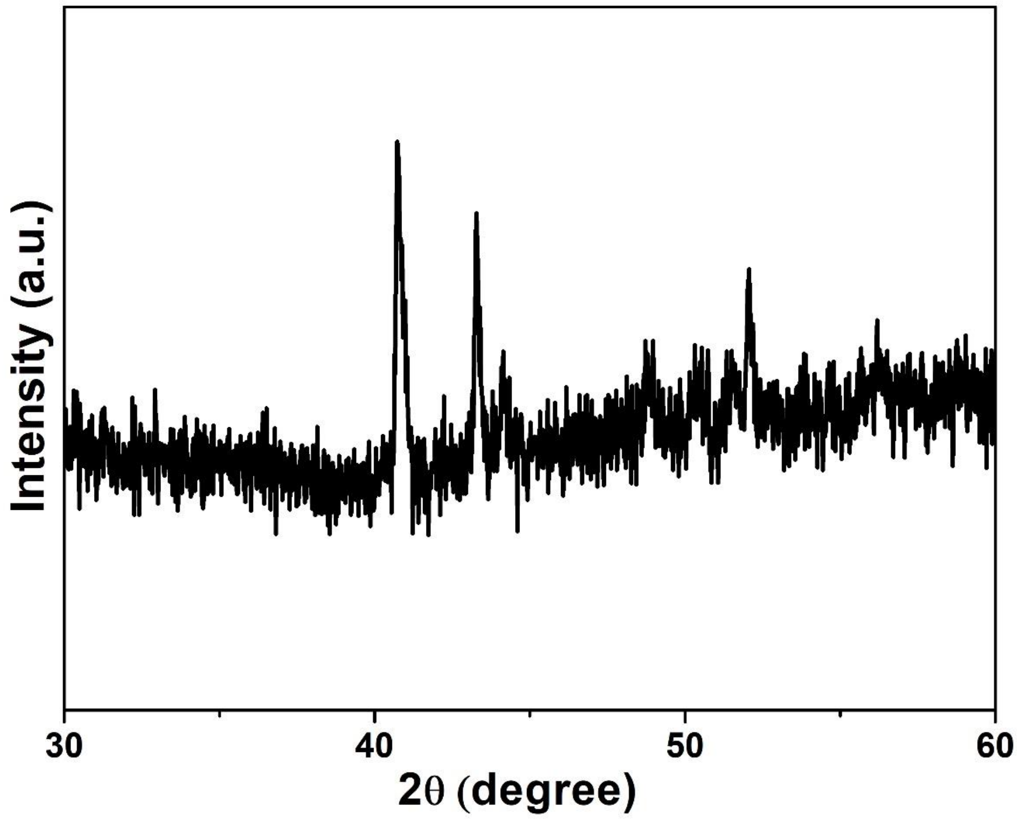


**Figure S2.** XRD pattern of $Co_2P$.

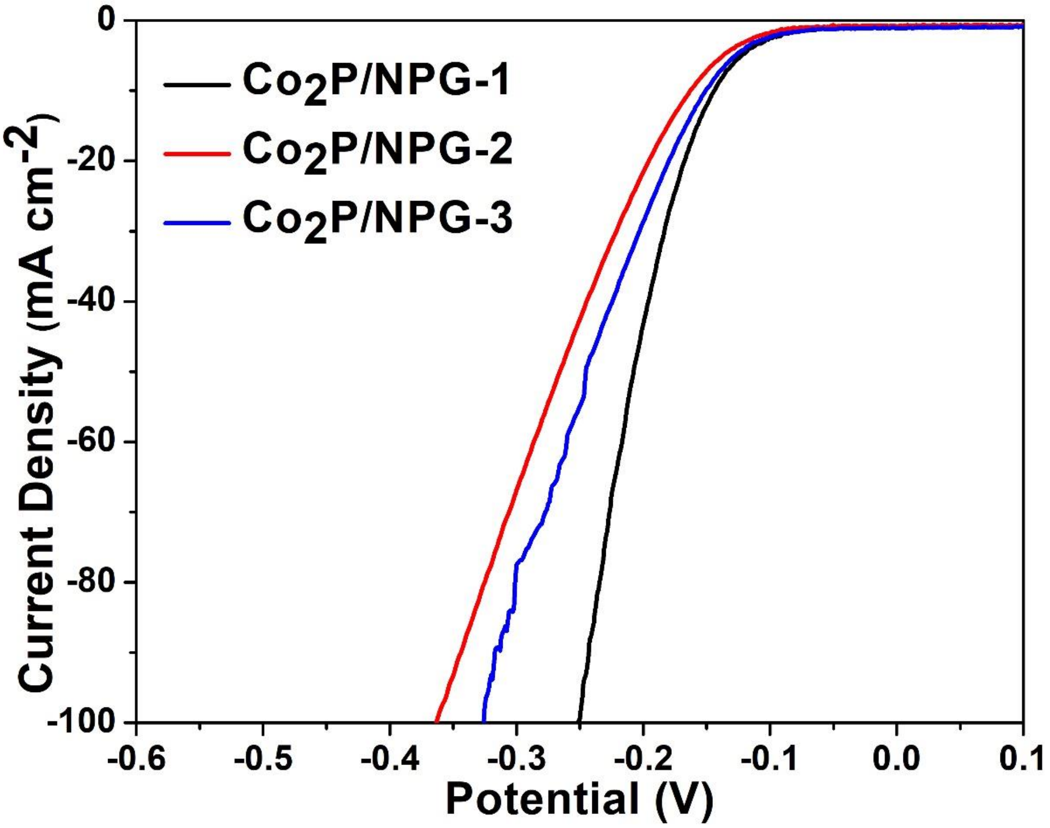


**Figure S3.** LSV curves of $Co_2P$/NPG synthesized with different contents of cobalt.

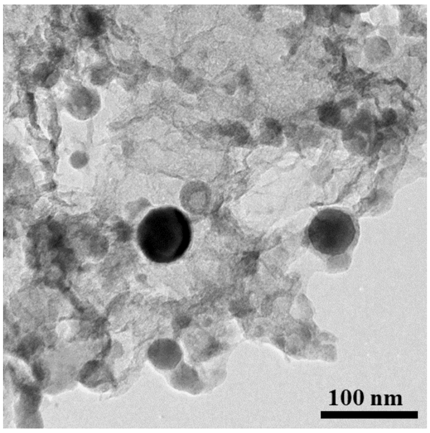


**Figure S4.** TEM image of $Co_2P$/NPG after stability test.
